# Modeling formalisms in Systems Biology

**DOI:** 10.1186/2191-0855-1-45

**Published:** 2011-12-05

**Authors:** Daniel Machado, Rafael S Costa, Miguel Rocha, Eugénio C Ferreira, Bruce Tidor, Isabel Rocha

**Affiliations:** 1 IBB-Institute for Biotechnology and Bioengineering/Centre of Biological Engineering, University of Minho, Campus de Gualtar, 4710-057 Braga, Portugal; 2 Department of Informatics/CCTC, University of Minho, Campus de Gualtar, 4710-057 Braga, Portugal; 3 Department of Biological Engineering/Computer Science and Artificial Intelligence Laboratory, Massachusetts Institute of Technology, Cambridge, MA 02139, USA

**Keywords:** Systems Biology, Modeling Formalisms, Biological Networks

## Abstract

Systems Biology has taken advantage of computational tools and high-throughput experimental data to model several biological processes. These include signaling, gene regulatory, and metabolic networks. However, most of these models are specific to each kind of network. Their interconnection demands a whole-cell modeling framework for a complete understanding of cellular systems. We describe the features required by an integrated framework for modeling, analyzing and simulating biological processes, and review several modeling formalisms that have been used in Systems Biology including Boolean networks, Bayesian networks, Petri nets, process algebras, constraint-based models, differential equations, rule-based models, interacting state machines, cellular automata, and agent-based models. We compare the features provided by different formalisms, and discuss recent approaches in the integration of these formalisms, as well as possible directions for the future.

## Introduction

Living organisms are complex systems that emerge from the fundamental building blocks of life. Systems Biology (SB) is a field of science that studies these complex phenomena currently, mainly at the cellular level (Kitano 2002). Understanding the mechanisms of the cell is essential for research in several areas such as drug development and biotechnological production. In the latter case, metabolic engineering approaches are applied in the creation of microbial strains with increased productivity of compounds with industrial interest such as biofuels and pharmaceutical products (Stephanopoulos 1998). Using mathematical models of cellular metabolism, it is possible to systematically test and predict manipulations, such as gene knockouts, that generate (sub)optimal phenotypes for specific applications (Burgard et al. 2003, Patil et al. 2005). These models are typically built in an iterative cycle of experiment and refinement, by multidisciplinary research teams that include biologists, engineers and computer scientists.

The interconnection between different cellular processes, such as metabolism and genetic regulation, reflects the importance of the holistic approach introduced by the SB paradigm in replacement of traditional reductionist methods. Although most cellular components have been studied individually, the behavior of the cell emerges at the network-level and requires an integrative analysis.

Recent high-throughput experimental methods generate the so-called *omics *data (e.g.: genomics, transcriptomics, proteomics, metabolomics, fluxomics) that have allowed the reconstruction of many biological networks (Feist et al. 2008). However, despite the great advances in the area, we are still far from a whole-cell computational model that integrates and simulates all the components of a living cell. Due to the enormous size and complexity of intracellular biological networks, computational cell models tend to be partial and focused on the application of interest. Also, due to the multidisciplinarity of the field, these models are based on several different kinds of formalisms, including those based on graphs, such as Boolean networks, and equation-based ones, such as ordinary differential equations (ODEs). This diversity can lead to the fragmentation of modeling efforts as it hampers the integration of models from different sources. Therefore, the whole-cell simulation goals of SB would benefit with the development of a framework for modeling, analysis and simulation that is based on a single formalism. This formalism should be able to integrate the entities and their relationships, spanning all kinds of biological networks.

This work reviews several modeling formalisms that have been used in SB, comparing their features and relevant applications. We opted to focus on the formalisms rather than the tools as they are the essence of the modeling approach. For the software tools implementing the formalisms, the interested reader may use the respective references. Note that besides the intracellular level, several studies in SB also address the cellular population level. Therefore, formalisms for modeling the dynamics of cellular populations that have received attention in the field were also considered in this work.

There are some interesting reviews already published in the literature. However they usually focus only on particular biological processes. An excellent review regarding the modeling of signaling pathways was elaborated by Aldridge et al. (2006). They address the model design process, as well as, model validation and calibration. They highlight the application of ODE and rule-based models, but do not mention other formalisms. Another recent review on the modeling of signaling networks can be found in Morris et al. (2010). Two remarkable reviews on the modeling of gene regulatory networks are presented by Schlitt and Brazma (2007) and by Karlebach and Shamir (2008). Both give examples of several applications of different formalisms for modeling this kind of networks. A few reviews with broader scope can also be found in the literature. Two excellent examples are Fisher and Henzinger (2007) and Materi and Wishart (2007). Both give a critical discussion on the application of different formalisms for computational modeling of cellular processes. The former covers Boolean networks, interacting state machines, Petri nets, process algebras and hybrid models, whereas the latter covers differential equations, Petri nets, cellular automata, agent-based models and process algebras. The lack of a single comprehensive review that compares a larger spectrum of formalisms motivated the development of this work.

## Biological Networks

**Figure 1:**
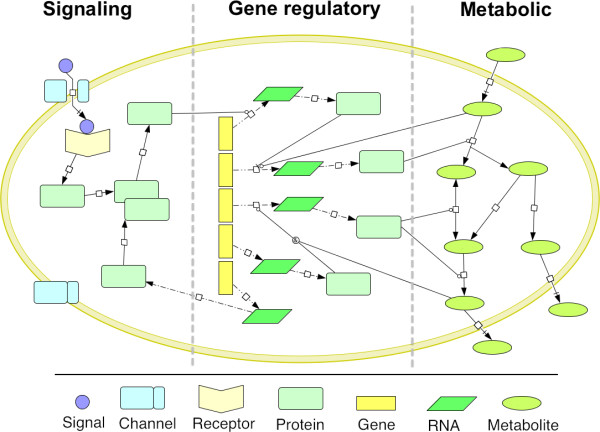
**The main cellular processes**. Conceptual representation of the main cellular processes that occur inside the cell. Signaling cascades receive external signals from the environment, either by binding to an extracellular receptor or, as illustrated, by passing through a channel and binding to an internal receptor. This signal is then propagated through a signaling cascade that involves the sequential phosphorylation of several proteins, leading to gene activations. Gene regulatory networks control the transcription level of genes. Genes are transcribed into RNA molecules, which are subsequently translated into proteins. These proteins are involved in all cellular functions. Some proteins are enzymes involved in the catalysis of metabolic reactions. Metabolic networks obtain energy and carbon from external sources using internal conversion steps. The internal metabolites can be used for cellular growth, or converted into by-products that are excreted by the cell. Their concentration level can also influence gene regulation.

Cells are composed by thousands of components that interact in a myriad of ways. Despite this intricate interconnection, it is usual to divide and classify these networks according to their biological function. A very simplistic example can be found in Figure 1 (created with the free software tool CellDesigner (Funahashi et al. 2003), that uses the graphical notations defined in (Kitano et al. 2005)). The main types of networks are signaling, gene regulatory and metabolic (although some authors also classify protein-protein interactions as another type of network).

### Signaling networks

Signal transduction is a process for cellular communication where the cell receives (and responds to) external stimuli from other cells and from the environment. It affects most of the basic cell control mechanisms such as differentiation and apoptosis. The transduction process begins with the binding of an extracellular signaling molecule to a cell-surface receptor. The signal is then propagated and amplified inside the cell through signaling cascades that involve a series of trigger reactions such as protein phosphorylation. The output of these cascades is connected to gene regulation in order to control cell function. Signal transduction pathways are able to crosstalk, forming complex signaling networks (Gomperts et al. 2009, Albert and Wang 2009).

### Gene regulatory networks

Gene regulation controls the expression of genes and, consequently, all cellular functions. Although all of the cell functionality is encoded in the genome through thousands of genes, it is essential for the survival of the cell that only selected functions are active at a given moment. Gene expression is a process that involves transcription of the gene into mRNA, followed by translation to a protein, which may be subject to post-translational modification. The transcription process is controlled by transcription factors (TFs) that can work as activators or inhibitors. TFs are themselves encoded by genes and subject to regulation, which altogether forms complex regulatory networks (Schlitt and Brazma 2007, Karlebach and Shamir 2008).

### Metabolic networks

Metabolism is a mechanism composed by a set of biochemical reactions, by which the cell sustains its growth and energy requirements. It includes several catabolic and anabolic pathways of enzyme-catalyzed reactions that import substrates from the environment and transform them into energy and building blocks required to build the cellular components. Metabolic pathways are interconnected through intermediate metabolites, forming complex networks. Gene regulation controls the production of enzymes and, consequently, directs the metabolic flux through the appropriate pathways in function of substrate availability and nutritional requirements (Steuer and Junker 2008, Palsson 2006).

## Modeling Requirements

Due to the different properties and behavior of the biological networks, they usually require different modeling features (although some desired features such as graphical visualization are common). For instance, features such as stochasticity and multi-state components may be important for signaling but not for metabolic networks. A summary of the major modeling features required by these networks is presented next.

### Network visualization

Biological models should be expressed as intuitively as possible and easily interpreted by people from different areas. For that matter, graph and diagram based formalisms can be more appealing than mathematical or textual notations. Such formalisms can take advantage of state of the art network visualization tools that, when compared to traditional textbook diagrams, allow a much better understanding of the interconnections in large-scale networks, as well as the integration of heterogeneous data sources (Pavlopoulos et al. 2008).

### Topological analysis

A considerable amount of the work in this field is based on topological analysis of biological networks. In this case, graph-based representations also play a fundamental role. The analysis of the topological properties of these graphs, such as degree distribution, clustering coefficient, shortest paths or network motifs can reveal crucial information from biological networks, including organization, robustness and redundancy (Jeong et al. 2000, Barabási and Oltvai 2004, Assenov et al. 2008).

### Modularity and hierarchy

Despite its great complexity, the cell is organized as a set of connected modules with specific functions (Hartwell et al. 1999, Ravasz et al. 2002). Taking advantage of this modularity can help to alleviate the complexity burden, facilitating the model analysis. Compositionality is a related concept meaning that two modeling blocks can be aggregated together into one model without changes to any of the submodels. This property can be of special interest for applications in Synthetic Biology (Andrianantoandro et al. 2006).

While modularity represents the horizontal organization of the cell, living systems also present vertical organization (Cheng and Hu 2010). Molecules, cells, tissues, organs, organisms, populations and ecosystems reflect the hierarchical organization of life. A modeling formalism that supports hierarchical models and different levels of abstraction will cope with models that connect vertical organization layers using top-down, bottom-up or middle-out approaches (Noble 2002).

### Multi-state components

Some compounds may have multiple states, for example, a protein may be modified by phosphorylation. This is a very common case in signaling networks. The state of a protein can affect its functionality and consequently the reactions in which it participates. Therefore, different states are represented by different entities. However, a protein with *n *binding sites will have 2^*n *^possible states, which results in a combinatorial explosion of entities and reactions (Hlavacek et al. 2003, Blinov et al. 2004). To avoid this problem, a suitable modeling formalism should consider entities with internal states and state-dependent reactions.

### Spatial structure and compartmentalization

On its lowest level, the cell can be seen as a bag of mixed molecules. However, this bag is compartmentalized and requires transport processes for some species to travel between compartments. Furthermore, in some compartments, including the cytosol, the high viscosity, slow diffusion and amount of molecules may not be sufficient to guarantee a spatial homogeneity (Takahashi et al. 2005). Spatial localization and concentration gradients are actually important mechanisms in biological processes such as morphogenesis (Turing 1952).

### Qualitative analysis

Experimental determination of kinetic parameters to build quantitative models is a cumbersome task. Furthermore, they are dependent on the experimental conditions, and there is generally no guarantee that the *in vitro *values will match the *in vivo *conditions (Teusink et al. 2000). Therefore, several models are only qualitative. Although these models do not allow for quantitative simulations, they allow us to ask qualitative questions about the system and to learn valuable knowledge. For instance, elementary mode analysis is used for calculating all possible pathways through a metabolic network (Schuster et al. 1999).

### Dynamic simulation

Dynamic simulation allows the prediction of the transient behavior of a system under different conditions. For each model, the particular simulation approach depends on the type of components included, which depend on the nature of the involved interactions and also on the available information for their characterization.

In regulatory networks, genes are activated and deactivated through the transcription machinery. Due to their complexity and the lack of kinetic information, the transcriptional details are usually not considered. Instead, genes are modeled by discrete (typically boolean) variables that change through discrete time steps. This is the simplest simulation method and requires models with very little detail.

Signaling cascades are triggered by a low number of signaling molecules. Therefore, it is important to take into consideration the inherent stochasticity in the diffusion of these molecules. Stochastic simulation is a common approach for simulation of signaling networks (Costa et al. 2009). This approach requires the attribution of probability functions for each reaction in the model.

Metabolic reactions, on the other hand, comprise large quantities of metabolites. Therefore, their behavior can be averaged and modeled by continuous variables governed by deterministic rate laws (Chassagnole et al. 2002). This requires a significant amount of experimental data for estimation of the kinetic parameters.

### Standardization

Biological models need to be represented in a common format for exchange between different tools. The Systems Biology Markup Language (SBML) has become the *de facto *standard of the SB community, and is currently supported by over two hundred tools (Hucka et al. 2003). It is an XML-based language for representation of species, compartments, reactions and their specific properties such as concentrations, volumes, stoichiometry and rate laws. It also facilitates the storage of tool specific data using appropriate tags. SBML was initially focused on biochemical reaction networks such as metabolic and signaling pathways, therefore it is not so well-suited for modeling other kinds of processes such as regulatory networks which are better described by logical models. Nevertheless, these and other limitations are being addressed in the development of future releases (Finney and Hucka 2003, Hucka et al. 2010).

CellML is another XML-based language with a similar purpose to SBML albeit more generic (Lloyd et al. 2004). The Systems Biology Graphical Notation (SBGN) (Le Novère et al. 2009) is a standard that focuses on the graphical notation and may be seen as a complement to SBML. It addresses the visualization concerns discussed previously, specially the creation of graphical models with a common notation that can be shared and unambiguously interpreted by different people.

## Modeling Formalisms

**Figure 2:**
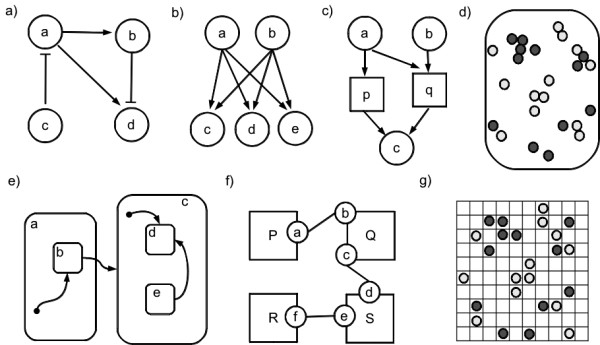
**Formalisms with visual representation **Toy examples of the formalisms with visual representation. a) Boolean network: genes are represented by nodes (*a*, *b*, *c*, *d*) and the arrows represent activation and repression; b) Bayesian network: the value of the output nodes (genes *c*, *d*, *e*) are given by a probability function that depends on the value of the input nodes (genes *a *and *b*); c) Petri net: places represent substances (*a*, *b*, *c*), transitions represent reactions (*p*, *q*) and the arrows represent consumption and production; d) Agent-based model: two types of agents, representing two different kinds of cells (or two kinds of molecules) can move freely and interact within the containing space; e) Interacting state machine: systems are represented by their state (*a*, *b*), where each state may contain one or more internal sub-states (*b*, *d*, *e*), arrows represent the transition between different states of the system; f) Rule-based model (represented by a contact map): agents represent proteins (*P*, *Q*, *R*, *S*), which may contain different binding sites (*a *to *f *), the connections represent the rules for possible interactions (such as phosphorylation); g) Cellular automata: a grid where the value of each element can represent different kinds of cells (or molecules), that can change by interaction with their immediate neighbors.

Many formalisms have been used to model biological systems, in part due to the diversity of phenomena that occur in living systems, and also due to the multidisciplinarity of the research teams. Biologists may be more familiar with mathematical modeling and computer scientists may be religious to their computational formalism of choice. The dichotomy between mathematical and computational models has been discussed elsewhere (Hunt et al. 2008). Although they follow different approaches (denotational *vs *operational), it has been questioned if there is such a clear separation between mathematical and computational models. Therefore, we will briefly describe several formalisms regardless of such distinction. Table 1 summarizes some of the literature references reviewed herein, classified by type of intracellular process implemented. Toy examples of the formalisms with graphical notation are depicted in Figure 2.

**Table 1:** Literature references grouped by formalism

	BN	Bay	PN	PA	CB	DE	RB	ISM	CA	AB
Signaling	+	+	++	++	+	++	++	++	+	++
Gene regulatory	++	++	+		+	++			+	
Metabolic			++		++	++			+	+

Overview of the amount of literature references for each formalism classified by the type of biological process. (+) Few references; (++) Several references; (BN) Boolean networks; (Bay) Bayesian networks; (PN) Petri nets; (PA) Process algebras; (CB) Constraint-based models; (DE) Differential equations; (RB) Rule-based models; (ISM) Interacting state machines; (CA) Cellular automata; (AB) Agent-based models.

### Boolean networks

Boolean networks (Figure 2a) were introduced by Kauffman in 1969 to model gene regulatory networks (Kauffman 1969). They consist on networks of genes, modeled by boolean variables that represent active and inactive states. At each time step, the state of each gene is determined by a logic rule which is a function of the state of its regulators. The state of all genes forms a global state that changes synchronously. For large network sizes (*n *nodes) it becomes impractical to explore all possible states (2^*n*^). This type of model can be used to find steady-states (called attractors), and to analyze network robustness (Li et al. 2004). Boolean networks can be inferred directly from experimental gene expression time-series data (Akutsu et al. 1999, D'haeseleer et al. 2000). They have also been applied in some studies to model signaling pathways (Gupta et al. 2007, Saez-Rodriguez et al. 2007). To cope with the inherent noise and the uncertainty in biological processes, stochastic extensions like Boolean networks with noise (Akutsu et al. 2000) and Probabilistic Boolean networks (Shmulevich et al. 2002) were introduced.

### Bayesian networks

Bayesian networks (Figure 2b) were introduced in the 80's by the work of Pearl (Pearl 1988). They are a special type of probabilistic graphs. Their nodes represent random variables (discrete or continuous) and the edges represent conditional dependencies, forming a directed acyclic graph. Each node contains a probabilistic function that is dependent on the values of its input nodes. There are learning methods to infer both structure and probability parameters with support for incomplete data. This flexibility makes Bayesian networks specially interesting for biological applications. They have been used for inferring and representing gene regulatory (Friedman 2004, Pena et al. 2005, Grzegorczyk et al. 2008, Auliac et al. 2008) and signaling networks (Sachs et al. 2002; 2005). One disadvantage of Bayesian networks is the inability to model feedback loops, which is a common motif in biological networks. This limitation can be overcome by dynamic Bayesian networks (Husmeier 2003, Kim et al. 2003, Zou and Conzen 2005, Dojer et al. 2006). In this case, the variables are replicated for each time step and the feedback is modeled by connecting the nodes at adjacent time steps.

### Petri nets

Petri nets (Figure 2c) were created in the 60's by Carl Adam Petri for the modeling and analysis of concurrent systems (Petri 1962). They are bipartite graphs with two types of nodes, *places *and *transitions*, connected by directed arcs. Places hold *tokens *that can be produced (respectively, consumed) when an input (respectively, output) transition *fires*. The execution of a Petri net is non-deterministic and specially suited for distributed systems with concurrent events. Their application to biological processes began in 1993, by the work of Reddy and coworkers, to overcome the limitations in quantitative analysis of metabolic pathways (Reddy et al. 1993).

There are currently several Petri net extensions (*e.g*.: coloured, timed, stochastic, continuous, hybrid, hierarchical, functional), forming a very versatile framework for both qualitative and quantitative analysis. Due to this versatility, they have been used in metabolic (Küffner et al. 2000, Zevedei-Oancea and Schuster 2003, Koch et al. 2005), gene regulatory (Chaouiya et al. 2004; 2008), and signaling networks (Sackmann et al. 2006, Chen et al. 2007, Breitling et al. 2008, Hardy and Robillard 2008). Also, they are suited for integrating different types of networks, such as gene regulatory and metabolic (Simao et al. 2005).

### Process algebras

Process algebras are a family of formal languages for modeling concurrent systems. They generally consist on a set of process primitives, operators for sequential and parallel composition of processes, and communication channels. The Calculus of Communicating Systems (CCS) was one of the first process algebras, developed during the 70's by Robin Milner (Milner 1980), and later gave origin to the more popular *π*-calculus (Milner et al. 1992). In SB the application of process algebras has been mainly focused on signaling pathways due to their similarity to communication processes. About a decade ago, Regev and coworkers published their pioneer work on the representation of signaling pathways with *π*-calculus (Regev et al. 2000; 2001). They later extended their work using stochastic *π*-calculus (BioSpi) to support quantitative simulations (Priami et al. 2001) and using Ambient calculus (BioAmbients) for representation of compartments (Regev et al. 2004). Other relevant biological applications of process algebras include Bio-calculus (Nagasaki et al. 1999), *κ*-calculus (for protein-protein interactions) (Danos and Laneve 2004), CCS-R (Danos and Krivine 2007), Beta binders (Priami and Quaglia 2005), Brane Calculi (Cardelli 2005), SpacePi (John et al. 2008), Bio-PEPA (Ciocchetta and Hillston 2008; 2009) and BlenX (Dematte et al. 2008, Priami et al. 2009).

### Constraint-based models

Constraint-based models for cellular metabolism began spreading during the 90's, mainly influenced by the work of Palsson and coworkers (Varma and Palsson 1994). Assuming that cells rapidly reach a steady-state, these models overcome the limitations in lack of experimental data for parameter estimation inherent in fully detailed dynamic models. They are based on stoichiometric, thermodynamic and enzyme capacity constraints (Reed and Palsson 2003, Price et al. 2003). Instead of a single solution, they define a space of possible solutions representing different phenotypes that comply with the constraints. The simplicity in this formulation allows its application to genome-scale metabolic models comprising thousands of reactions, such as the most recent metabolic reconstruction of *E. coli *(Orth et al. 2011).

Constraint-based models have been used in metabolic engineering strategies for the determination of flux distributions (metabolic flux analysis (Wiechert 2001), flux balance analysis (Kauffman et al. 2003)), knockout phenotype predictions (minimization of metabolic adjustment (Segrè et al. 2002), regulatory on/off minimization (Shlomi et al. 2005)) or enumerating all possible pathways (extreme pathways (Schilling et al. 2000), elementary flux modes (Schuster et al. 1999)). Although their main application has been on metabolic networks, there are recent efforts towards application on gene regulatory and signaling networks (Papin et al. 2005, Gianchandani et al. 2009, Lee et al. 2008a).

### Differential equations

Differential equations describe the rate of change of continuous variables. They are typically used for modeling dynamical systems in several areas. Systems of non-linear ordinary differential equations (ODEs) have been used in SB to describe the variation of the amount of species in the modeled system as a function of time. They have been applied to all kinds of biological pathways (Chassagnole et al. 2002, Tyson et al. 2003, Chen et al. 1999, Rizzi et al. 1997). With a fully detailed kinetic model, one can perform time-course simulations, predict the response to different inputs and design system controllers. However, building ODE models requires insight into the reaction mechanisms to select the appropriate rate laws, and experimental data to estimate the kinetic parameters. The lack of kinetic data has limited the size of the modeled networks to pathway size, with exception for the human red blood cell model (Jamshidi et al. 2001).

Approximative rate laws such as generalized mass action (GMA) (Horn and Jackson 1972), S-systems (Savageau and Voit 1987), lin-log (Visser and Heijnen 2003), and convenience kinetics (Liebermeister and Klipp 2006), have compact standard formulations that can facilitate the development and analysis of large-scale models (Heijnen 2005, Costa et al. 2010). This opens the possibility for kinetic modeling at the genome-scale (Smallbone et al. 2010).

Other types of differential equations, such as stochastic differential equations (SDEs) and partial differential equations (PDEs) can be used respectively to account for stochastic effects and spatial distribution (Turner et al. 2004). Piecewise-linear differential equations (PLDEs) have been used to integrate discrete and continuous features in gene regulatory networks (De Jong et al. 2004, Batt et al. 2005).

### Rule-based models

Rule-based (Figure 2f) modeling comprises a recent approach to the problem of multi-state components in biological models. In rule-based formalisms the species are defined in a structured manner and support multiple states. The reaction rules are defined as transformations of classes of species, avoiding the need for specifying one reaction per each possible state of a species. This high-level specification is then automatically transformed into a biochemical network with the set of species and reactions generated by the specification. This kind of formalism is implemented in BioNetGen (Blinov et al. 2004) which generates an ODE model or a stochastic simulation from the ruled-based specification. It has been applied in the modeling of different signaling pathways (Blinov et al. 2006, Barua et al. 2007; 2008; 2009). A similar rule-based formalism used for this kind of pathways is the *κ *language, where the species are defined by agents that have a structured interface for interaction with other agents (Danos et al. 2007; 2009, Feret et al. 2009). The possible interactions are defined by a set of rules, which can be visualized by a contact map. BIOCHAM implements a rule-based approach for model specification which is complemented with a temporal logic language for the verification of the properties the biological models (Calzone et al. 2006).

The main advantage of the rule-based approach is that it can avoid the combinatorial explosion problem in the generation and simulation of the complete reaction network by performing stochastic simulations that only instantiate the species and reactions as they become available (Colvin et al. 2009; 2010) or by the generation of coarse-grained ODE systems (Feret et al. 2009). Spatial simulation has been addressed recently by the inclusion of geometric information as part of the structure of the species (Gruenert et al. 2010).

### Interacting state machines

Interacting state (Figure 2e) machines are diagram-based formalisms that describe the temporal behavior of a system based on the changes in the states of its parts. They are suited to model biological behavior in a qualitative way as they require little quantitative data. They differ from other approaches as they define a system in terms of its states rather than its components. They are typically used for model checking and interactive execution.

One such formalism is Statecharts, developed by David Harel during the 80's (Harel 1987) that was first applied in biology for modeling the T-cell activation process (Kam et al. 2001, Efroni et al. 2003) and more recently in pancreatic organogenesis (Setty et al. 2008). In this formalism, the state of a system may contain sub-states at multiple levels, allowing an hierarchical view of the system and the relation between events at smaller and larger scales. Other related formalisms are Reactive Modules (Alur and Henzinger 1999) and Live Sequence Charts (Damm and Harel 2001), which, along with the former, have been applied in the modelling of *C. elegans *vulval development (Fisher et al. 2005; 2007).

### Cellular automata

Cellular automata (Figure 2g) were created by von Neumann and Ulam in the 40's (Von Neumann and Burks 1966). They are discrete dynamic models that consist on a grid of cells with a finite number of states. A cellular automaton has an initial configuration that changes at each time step through a predefined rule that calculates the state of each cell as a function of the state of its neighbors at the previous step. They are specially suited for modeling complex phenomena in a scale-free manner and have been used in biological studies for a long time (Ermentrout and Edelstein-Keshet 1993). Due to their spatial features their main applications are related to molecular dynamics and cellular population dynamics.

Application examples at the molecular level include enzyme reaction networks that account for spatial diffusion (Weimar 2002) and signaling pathways (Wurthner et al. 2000, Kier et al. 2005). At the cellular level they were used for models such as those of bacterial aggregation (Sozinova et al. 2005) and HIV infection (Zorzenon dos Santos and Coutinho 2001, Corne and Frisco 2008). Dynamic cellular automata are a variation of cellular automata that allows for movement of the cell contents inside the grid, mimicking brownian motion. They were used to model enzyme kinetics, molecular diffusion and genetic circuits (Wishart et al. 2005).

### Agent-based models

Agent-based models (Figure 2d) describe the interactions among multiple autonomous agents. They are similar in concept to cellular automata, except in this case, instead of using a grid and synchronized time steps, the agents move freely within the containing space. Likewise, they are used to study complex phenomena and emergent dynamics using populations of agents with simple rules. At the molecular level they have been mainly used to build models of signaling pathways that account for spatial distribution and the structural properties of the cell (Gonzalez et al. 2003, Pogson et al. 2006; 2008, An 2009). Recently, they have also been applied to metabolic reactions (Klann et al. 2011). However, their main application is at the multi-cellular level, where they have been used to study granuloma formation (Segovia-Juarez et al. 2004), tumor growth (Zhang et al. 2007, Engelberg et al. 2008), morphogenesis (Grant et al. 2006), chemotaxis (Emonet et al. 2005), immune responses (Lollini et al. 2006, Li et al. 2008), and several others (Thorne et al. 2007, Merelli et al. 2007).

### Other formalisms

There are other modeling formalisms that have been used in SB which are worth mentioning. Cybernetic modeling is one of the earliest approaches for dynamic modeling that was used in bioprocess applications (Kompala et al. 1984, Dhurjati et al. 1985). A recent approach combines cybernetic variables with elementary flux modes (Young et al. 2008, Kim et al. 2008). Hybrid automata addressed the integration of discrete and continuous components in the Delta-Notch signaling pathway (Ghosh and Tomlin 2001; 2004). Artificial neural networks were used to model gene expression (Vohradsky 2001). Molecular interaction maps are a popular graph-based formalism created by Kohn in 1999 (Kohn 1999, Kohn et al. 2006, Luna et al. 2011) that influenced the SBGN standard (Le Novère et al. 2009). Other graph-based formalisms include modular interaction networks (Yartseva et al. 2007) and logical interaction hypergraphs (Klamt et al. 2006). The P systems formalism created by Paun in 1998, inspired the area of membrane computing (Paun 2000) and has been recently applied in SB (Pérez-Jiménez and Romero-Campero 2006, Cao et al. 2010). Chemical organization theory is a recent approach for modeling biochemical reaction networks that uses set theory to analyze how they can be decomposed into self-maintaining subnetworks called organizations, that reveal dynamic properties of the system (Dittrich and Di Fenizio 2007). It has been used to analyze different types of networks including signaling pathways and regulated metabolic networks (Centler et al. 2007; 2008, Kaleta et al. 2008; 2009).

### Formalisms conversion

The inability of the formalisms to fit all purposes has driven the development of methodologies to convert between different formalisms. Two different methods have been proposed to convert Boolean networks to Petri nets (Chaouiya et al. 2004, Steggles et al. 2007). Boolean networks have also been converted to constraint-based models (Gianchandani et al. 2006) and to ODEs (Wittmann et al. 2009). Other formalisms have also been converted to ODEs, including constraint-based models (Smallbone et al. 2007), Petri nets (Gilbert and Heiner 2006), process algebras (Calder et al. 2005) and rule-based models (Feret et al. 2009). When the mappings are made from abstract to more detailed models they usually require some assumptions and insight into the reaction mechanisms. The language for biochemical systems (LBS) is a recent language that integrates a rule-based approach with process calculus, and supports the generation of Petri nets, ODEs and continuous time Markov chains (Pedersen and Plotkin 2010).

### Formalisms integration

Along with the conversion between formalisms, there is also a recent trend for developing methods that support integrated simulation of different formalisms in order to integrate different kinds of biological networks, where each network is modeled in its own formalism. Extensions of flux balance analysis (FBA) (Kauffman et al. 2003), such as regulated FBA (rFBA) (Covert and Palsson 2002) and steady-state regulated FBA (SR-FBA) (Shlomi et al. 2007) incorporate boolean rules into constraint-based models for integrated simulation of regulatory and metabolic networks. Integrated FBA (iFBA) extends rFBA by integrating kinetic information from ODE models (Covert et al. 2008). Integrated dynamic FBA (idFBA) aims to integrate signaling, regulatory and metabolic networks by modeling all networks in the constraint-based formulation (Lee et al. 2008b). Biochemical systems theory (BST) has been recently integrated with Hybrid Functional Petri Nets (HFPN) in order to integrate metabolic, regulatory and signaling networks, in a framework that accounts for different time-scales as well as discrete, stochastic and continuous effects (Wu and Voit 2009a;b).

### Comparison of the Formalisms

The diversity of problems studied in SB gave rise to the application of several different types of formalisms. A comparison of the amount of literature references for each formalism, classified by the type of biological process described, is given in Table 1. We can observe that only four formalisms (Petri nets, constraint-based models, differential equations and cellular automata) have been applied to all three types of biological networks, which makes them potential candidates as a suitable integrative formalism for whole-cell modeling. However, this should not exclude other formalisms from this possibility as well. Another interesting observation is that metabolism is the biological process with the smaller number of formalisms applied. This is likely due to the fact that its two main frameworks (differential equations and constraint-based) are well suited for modeling metabolic networks. On the other hand, all of the formalisms have been applied to signaling pathways. One possible reason is that they require the largest number of modeling features, including spatial localization and multi-state components.

The modeling features provided by the formalisms reviewed in this work are compared in Table 2. Some of the features are only available in extensions of the formalisms. We can observe that no single formalism covers the whole spectrum of features desired for modeling all kinds of biological components. Petri nets and rule-based models are among the formalisms that cover most features. Petri nets have several extensions available, and although none of the extensions alone fulfills all requisites, altogether they form a very versatile modeling framework. Rule-based models present a high level of abstraction and can be used for stochastic simulation and automatic generation of lower level ODE-based representations. Therefore, they take advantage of the analytic power of abstract representations, preserving the ability to generate stochastic and deterministic simulations.

**Table 2:** Modeling formalisms and implemented features

	BN	Bay	PN	PA	CB	DE	RB	ISM	CA	AB
Visualization	+	+	+				+	+	+	+
Topology	+	+	+		+					
Modularity			+	+			+	+		
Hierarchy			e	e				+		
Multi-state			e				+	+	+	+
Compartments				e			+	+		+
Spatial						e	e		+	+
Qualitative	+	+	+	+	+		+			
Synchronized	+		e						+	
Stochastic	e	+	e	+		e	+	+	+	+
Continuous			e			+	+			

Modeling formalisms and implemented features. (+) Supported feature; (e) Available through extension; (BN) Boolean networks; (Bay) Bayesian networks; (PN) Petri nets; (PA) Process algebras; (CB) Constraint-based models; (DE) Differential equations; (RB) Rule-based models; (ISM) Interacting state machines; (CA) Cellular automata; (AB) Agent-based models.

Although none of the formalisms implements all the required features, this is not necessarily a limitation, since different formalisms can be used at different stages of the modeling process. The model construction process begins with biochemical knowledge and experimental data that allow an enumeration of the components and connections in the system. Graph-based models, such as Boolean networks, Bayesian networks and Petri nets can be used for modeling this map of interactions. This allows a deeper understanding of the organization of the system through topological analysis, and drives new experiments by finding gaps in the models. This kind of models also allows qualitative descriptions of system behavior and coarse simulation capabilities. If the reactions' stoichiometry and directionality are known, one may analyze the steady-states of the system using constraint-based models. Finally, if extensive experimental data is available to infer the kinetics of the reactions, probabilistic or deterministic rate laws can be used to create dynamic models. These are used to generate time-course simulations under different sets of initial conditions. Stochastic process algebras, stochastic Petri nets, continuous Petri nets, rule-based models and differential equations, would all be ideal candidates for this purpose.

Cellular automata and agent-based models account for the individual replicas of each component in the system. When applied at the molecular level, this paradigm provides accurate simulations of small sets of biochemical reactions that account for spatial diffusion. However, it becomes infeasible to perform simulations at the genome-scale network level, as this would imply modeling every copy of all substances present in the cell. Nevertheless, this approach is very convenient for modeling at the cell population level, as it allows to track changes in individual cells and to study the emergent properties of cellular communities.

In search for a proper formalism perhaps the most important aspect to consider is the balance between simplicity and expressiveness. There is a price to pay for the amount of features provided by a formalism, which may come at the cost of increased model complexity. The complexity of the representation and the number of parameters determines the amount of experimental data required for model construction. This is the reason why the most simple formalisms such as Boolean networks and constraint-based models have been used to build, respectively, gene regulatory and metabolic networks at the genome scale. This concern is most critical when not only the parameters but also the network structure are unknown. Model inference (also known as *reverse engineering*) methods are applied in these cases. They have been used to infer Boolean networks (Akutsu et al. 1999, D'haeseleer et al. 2000), Bayesian networks (Friedman 2004, Auliac et al. 2008), Petri nets (Nummela and Julstrom 2005, Durzinsky et al. 2011) and ODEs (Kimura et al. 2005, Iba 2008) from experimental data. However, the scalability of these methods is greatly dependent on the simplicity of the underlying formalism.

### Perspective

With the myriad of formalisms that have been applied in SB, we face the challenge of choosing the proper formalism for the problem in hands. As more data become available for network reconstruction, we move towards integration of all kinds of biological networks, namely signaling, gene regulatory and metabolic. Although some formalisms like Petri nets, constraint-based models and differential equations have been applied for all these networks, no single formalism covers the whole spectrum of functionalities reviewed in this work. Petri nets have several extensions available, covering most of the features analyzed, with the exception of compartments and spatial localization. Rule-based models are another strong candidate as they also cover a great part of the modeling features. These are definitely two formalisms to keep under consideration in the near future.

The model building process is based on iterative steps of refinement and validation. Recent approaches for genome-scale kinetic modeling of metabolism, begin with the network topology, modeled in the constraint-based framework, and then refine the models by adding the kinetic structure in order to generate ODE models (Jamshidi and Palsson 2010, Smallbone et al. 2010). Petri nets seem to be a promising formalism for this purpose, given that discrete Petri nets can model the network topology, and can then be used as a scaffold for the generation of dynamic models based on continuous or stochastic Petri nets. The fact that the same kind of formalism is used during the whole model refinement process, helps the creation of more straightforward methods for automatic mapping and validation of the models.

A common problem in the analysis of biological networks is the combinatorial explosion that originates from the complexity of large models. A typical example is the computation of elementary flux modes at the genome-scale, requiring modular decomposition of the networks (Schuster et al. 2002). This problem will aggravate as we get closer to whole-cell modeling. The solution may reside in the application of hierarchical formalisms to represent an intermediate level between the reaction and the cell. As stated elsewhere, one should not "model bulldozers with quarks" (Goldenfeld 1999). Hierarchical Petri nets, BioAmbients and Statecharts are formalisms that support hierarchical modeling.

Models of cell populations are also becoming more frequent. They are used to study scenarios like cell differentiation, chemotaxis, infections or tumor growth. This kind of models depends on the internal dynamics of the cells as well as population dynamics. Therefore, they require modeling of interactions across organizational scales (Walker and Southgate 2009). It is possible that in the future, we will have multi-scale models that integrate formalisms. For instance, the evolution of a population of cells may be modeled by an agent-based model, where each agent has a boolean network for internal representation of its gene expression.

In order to convert between different formalisms it is important to have a standard representation format that preserves most of the features in the models. SBML is the most popular standard in the SB community, currently supported by over two hundred tools (Hucka et al. 2003). Most of the modeling features covered herein have been proposed for future versions of SBML (Finney and Hucka 2003). These include hierarchical model composition, rule-based modeling, spatial geometry and alternative mathematical representations. The compatibility with the SBML representation will dictate which formalisms will prevail in the future.

Many of the proposed formalisms, such as Petri nets or process algebras, were originally created by the computational community for the specification of software systems, where the final system has to comply to the model. The biological community faces the opposite problem, where the model has to mimic the system's behavior, and where most components cannot even be measured directly. Therefore, a proper framework for SB must provide not only a suitable formalism with attractive features and simulation methods, but also methods for model inference and parameter estimation that are sufficiently robust to handle experimental data that are incomplete and prone to measurement error.

## Competing interests

The authors declare that they have no competing interests.
